# Primary health care for children - evidence for prevention

**DOI:** 10.1186/s12887-021-02787-w

**Published:** 2021-09-08

**Authors:** Susanne Carai, Martin W. Weber

**Affiliations:** 1 WHO, Regional office for Europe, Copenhagen, Denmark; 2 Witten/Herdecke University, Witten, Germany

Primary health care is the cornerstone of universal health coverage and guarantor of quality comprehensive health care for all, including children [1]. Views on what comprehensive primary health care entails and what health services can and should deliver at the primary health care level for children and adolescents differ. Different health systems offer a range of curative and preventive interventions for children and adolescents. Some of those interventions are evidence-based with proven benefits. Others are common practices based on tradition, expert opinion, or common sense and there is no felt need for justifying their effectiveness or benefit. Some interventions however may not only be without benefit for the child but harmful [2, 3].

**Fig. 1:**
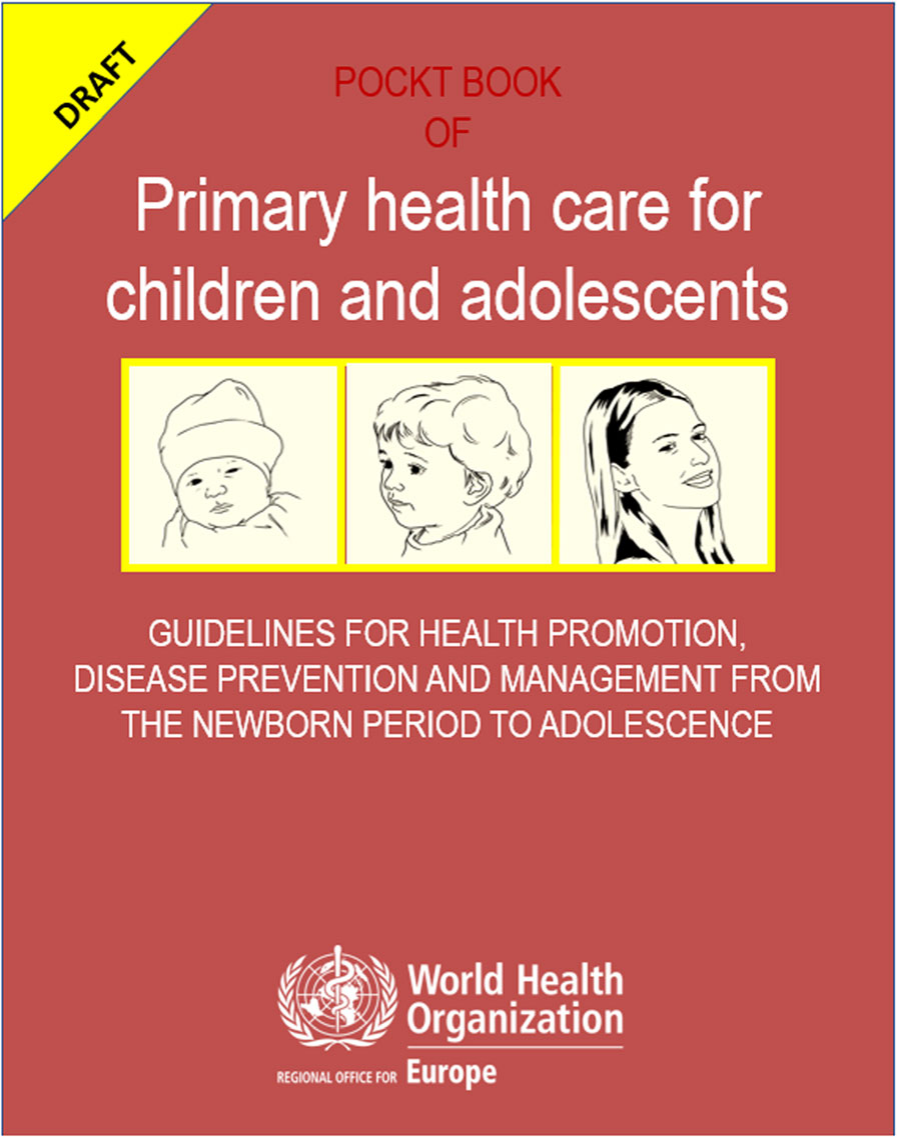
The draft WHO Pocket Book of Primary Health Care for Children and Adolescents, expected publication date end 2021

Against this backdrop, the WHO *Pocket Book on Primary Health Care for Children and Adolescents* (see Fig. 1 below) defines evidence-based standards of health care for a child or adolescent according to which every child attending primary health care services across the European Region should be cared for. During the development process of the *Pocket Book*, it became apparent that recommendations and practices for preventive interventions for infants and children are particularly controversial and the book’s editorial group and members of the *European* Confederation for Primary Care Paediatricians (ECPCP) realised the need for collating existing evidence for recommendations and practices for disease prevention for infants, children and adolescents.

This supplement summarises and reviews the available evidence for selected preventive interventions to inform key chapters of the *Pocket Book* and thereby helps to close the practice gap for prevention*.* In a series of articles, Sophie Jullien and colleagues first provide the background, describe the methods in detail, and present the supplement’s main findings [4]. The author then critically reviews and summarises the strengths of the recommendations and quality of evidence for ten preventive interventions based on major documents of international paediatric societies and institutions, mostly from Europe [5–14].

The *Pocket Book* aims to help deliver on the promise of primary health care: its focus on evidence-based practices and prevention should counteract inappropriate medicalisation of health services (i.e. preferring invasive or active treatment over conservative or watchful management, intravenous treatment over oral rehydration therapy and multiple drugs over just one) and prevent unnecessary treatment and hospitalisation. Its success, however, will also require a critical review of health-system financing.

As of today, health spending overwhelmingly goes towards curative care, not prevention: Europe’s governments spend only 3% of the total health expenditure (THE) on health promotion and disease prevention [15]. Funding needs to be made available to enable the provision of evidence-based treatment and prevention services for children and adolescents. These services need to be included in state-guaranteed benefits packages or covered under health insurance schemes to be made available for children and adolescents without cost to them at the point of care.

Attaining the sustainable development goals (SDGs) requires a substantial shift in thinking about child and adolescent health, according to the global review of the implementation of the UNICEF/WHO Strategy of the Integrated Management of Childhood Illness (IMCI) to reduce childhood mortality and improve quality of care for children [16]. The focus moves from the survival of children under 5 years old to a holistic view of child and adolescent health and attention shifts to health promotion, disease prevention, early risk factor management and monitoring of chronic conditions [17]. The summaries of evidence for preventive interventions for children published in this supplement are an important step in this direction and providing comprehensive evidence-based primary health care for children and adolescents.
